# Hi-TARGET: a fast, efficient and versatile CRISPR type I-B genome editing tool for the thermophilic acetogen *Thermoanaerobacter kivui*

**DOI:** 10.1186/s13068-025-02647-0

**Published:** 2025-04-30

**Authors:** Angeliki Sitara, Rémi Hocq, Alexander Jiwei Lu, Stefan Pflügl

**Affiliations:** 1 Institute for Chemical, Environmental and Bioscience Engineering, Technische Universität Wien, Gumpendorfer Straße 1a, 1060 Vienna, Austria; 2 Christian Doppler Laboratory for Optimized Expression of Carbohydrate-Active Enzymes, Technische Universität Wien, Gumpendorfer Straße 1a, 1060 Vienna, Austria

**Keywords:** Genome editing, Endogenous CRISPR/Cas system, Thermophilic acetogen, Gas fermentation, Metabolic engineering

## Abstract

**Background:**

Due to its ability to grow fast on CO_2_, CO and H_2_ at high temperatures and with high energy efficiency, the thermophilic acetogen *Thermoanaerobacter* *kivui* could become an attractive host for industrial biotechnology. In a circular carbon economy, diversification and upgrading of C1 platform feedstocks into value-added products (e. g., ethanol, acetone and isopropanol) could become crucial. To that end, genetic and bioprocess engineering tools are required to facilitate the development of bioproduction scenarios. Currently, the genome editing tools available for *T. kivui* present some limitations in speed and efficiency, thus restricting the development of a powerful strain chassis for industrial applications.

**Results:**

In this study, we developed the versatile genome editing tool Hi-TARGET, based on the endogenous CRISPR Type I-B system of *T. kivui*. Hi-TARGET demonstrated 100% efficiency for gene knock-out (from both purified plasmid and cloning mixture) and knock-in, and 49% efficiency for creating point mutations. Furthermore, we optimized the transformation and plating protocol and increased transformation efficiency by 245-fold to 1.96 × 10^4^ ± 8.7 × 10^3^ CFU μg^−1^. Subsequently, Hi-TARGET was used to demonstrate gene knock-outs (*pyrE*, *rexA*, *hrcA*), a knock-in (*ldh*::pFAST), a single nucleotide mutation corresponding to PolC^C629Y^, and knock-down of the fluorescent protein pFAST. Analysis of the ∆*rexA* deletion mutant created with Hi-TARGET revealed that the transcriptional repressor *rexA* is likely involved in the regulation of the expression of lactate dehydrogenase (*ldh*). Following genome engineering, an optimized curing procedure for edited strains was devised. In total, the time required from DNA to a clean, edited strain is 12 days, rendering Hi-TARGET a fast, robust and complete method for engineering *T. kivui*.

**Conclusions:**

The CRISPR-based genome editing tool Hi-TARGET developed for *T. kivui* can be used for scarless deletion, insertion, point mutation and gene knock-down, thus fast-tracking the generation of industrially-relevant strains for the production of carbon-negative chemicals and fuels as well as facilitating studies of acetogen metabolism and physiology.

**Supplementary Information:**

The online version contains supplementary material available at 10.1186/s13068-025-02647-0.

## Background

Thermoanaerobes are a group of facultative and obligate anaerobic, thermophilic bacteria and archaea which potentially offer advantages for industrial bioprocesses, as they combine the benefits of anaerobes (high carbon/energy efficiency) with those of the thermophiles (low contamination risk, lower cooling costs, relative ease of recovery of volatile products) [1]. In addition, thermoanaerobes can be particularly interesting for a circular carbon economy, due to their ability to grow on renewable and sustainable feedstocks such as syngas (obtained from biomass gasification or via electrolysis of captured atmospheric CO_2_) or lignocellulosic biomass [1].

The thermophilic acetogen *Thermoanaerobacter kivui* potentially offers a plethora of advantages for industrial bioprocesses, as it can grow fast on CO_2_ in cheap mineral medium without yeast extract or vitamin supplements, with high specific growth rates and high substrate turnover rates [2–7]. *T. kivui* utilizes the Wood-Ljungdahl pathway, the most efficient CO_2_ fixation pathway, to reduce one carbon compounds (C1) such as CO_2_ (+ H_2_), CO and formate to acetate, rendering the strain interesting for C1 bioprocessing [6–9]. Other heterotrophic substrates include glucose, mannose, mannitol, fructose, and pyruvate [10, 11]. While *T. kivui* has been shown to produce H_2_ and formate [12], the main product is acetate. Metabolic engineering is therefore required to expand and diversify the product spectrum of *T. kivui* to other value-added products (e.g., ethanol, lactate, acetone, 2,3-butanediol). Consequently, a powerful genome editing tool is required to facilitate the introduction of heterologous pathways for non-natural products, as well as to increase the bioenergetics for more energy-demanding products [1, 13].

Currently, the genetic toolbox of *T. kivui* features natural transformation with linear [9, 14] and circular DNA, the pMU131 replicative shuttle vector [15], a fluorescent reporter system based on the thermostable pFAST marker [16], characterized inducible promoters for controlling gene expression [14], and a genome editing method based on the selectable/counter-selectable marker *pyrE* [15]. The latter technique has been used in recent years to study the function of various genes in *T. kivui*, mostly through gene deletion [6, 15, 17, 18]. The *pyrE* system is a two-step process based on homologous recombination (HR), and on a dual selectable phenotype arising in cells bearing (prototrophy for uracil) or lacking *pyrE* (resistance to 5’- fluoroorotic acid, 5’-FOA). However, the technique is labor-intensive, requiring multiple sub-culturing, selection, and screening steps, and possibly inefficient as the second crossover can either produce the desired mutant or restore a wild-type genotype [19]. Therefore, the development of a fast and efficient genome editing tool is crucial for advancing our understanding of *T. kivui* physiology and facilitating the development of industrial strains.

In recent years, CRISPR/Cas-based genome editing techniques have been developed for a broad range of hosts (bacteria, archaea, fungi, yeast, mammalian cells) as they allow for efficient, precise, versatile and multiplex genome editing [20]. During CRISPR-based genome editing, Cas proteins form a complex with the CRISPR RNA (crRNA), target a sequence (protospacer) downstream of the protospacer-adjacent motif (PAM) and create a double-strand break (DSB), that most bacteria cannot easily repair, resulting in cell death [21, 22]. When a repair template is present (homologous recombination arms, HAs), the cells are rescued and genetically modified, with CRISPR efficiency depending on direct double crossover selection and thus, preventing reversion to the wild type, as is the case for the *pyrE* method [22].

In the type I-B CRISPR system, Cas1 and Cas2 are responsible for acquiring new spacers from invading genetic elements*.* Cas6 processes the transcribed pre-CRISPR RNA (pre-crRNA), by cleaving it into repeat-protospacer-repeat segments, and generates the mature crRNA [23]. Cas4 interacts with Cas1 and Cas2 to identify invading elements by ensuring compatibility with the PAM [24]. The cascade proteins (Cas5–8) form a complex with the crRNA, recognize the PAM, and recruit Cas3, the type I-B nuclease, which initially creates a single-stranded DNA break (SSB) and eventually a DSB in the target sequence [22].

The application of the endogenous type I-B CRISPR system for genome editing has been demonstrated in many bacteria, including thermophiles such as *Thermoanerobacterium aotearoense* SCUT27, *Acetivibrio thermocellus* (formerly *Clostridium thermocellum*) and *Parageobacillus thermoglucosidasius* with efficiencies ranging between 30 and 100% [19, 25–29]. In cases where gene deletion is not possible—as is the case for essential genes [30]—CRISPR interference (CRISPRi) can be an alternative method for regulating gene expression. Typically, a mutated Cas9 (dCas9) is used for CRISPRi, binding to DNA sequences and modulating their expression [31]. However, an alternative approach that utilizes the endogenous system was demonstrated in *Haloarcula hispanica* and *P. thermoglucosidasius* [32, 33]*.* This method employs a shortened, non-canonical crRNA (creA), which partially matches the target DNA and, together with the cascade, partially inhibits transcription [33].

In this work, we developed the novel CRISPR/Cas genome editing tool Hi-TARGET (High-efficiency *Thermoanaerobacter kivui* CRISPR genome editing tool) for *T. kivui* based on the endogenous type I-B CRISPR machinery. With an improved transformation protocol and the CRISPR genome editing system, we performed three gene knock-outs (Δ*pyrE,* Δ*rexA,* Δ*hrcA*), a knock-in (*ldh*::pFAST), a single nucleotide mutation (PolC^C629Y^), and showcased knock-down of the fluorescent protein pFAST in *T. kivui*. We demonstrate the versatility of Hi-TARGET as it allows for fast and efficient genome editing – succeeding even by directly using the cloning mixture as DNA input. Furthermore, successful curing of the genome-edited strains for subsequent editing completes the genetic engineering suite for *T. kivui*, which is required to fast-track the development of new edited strains that will promote understanding of acetogen metabolism but also accelerate the production of sustainable chemicals with *T. kivui*.

## Results and discussion

### *Thermoanaerobacter kivui* bears a functional Type I-B CRISPR system

**Fig. 1:**
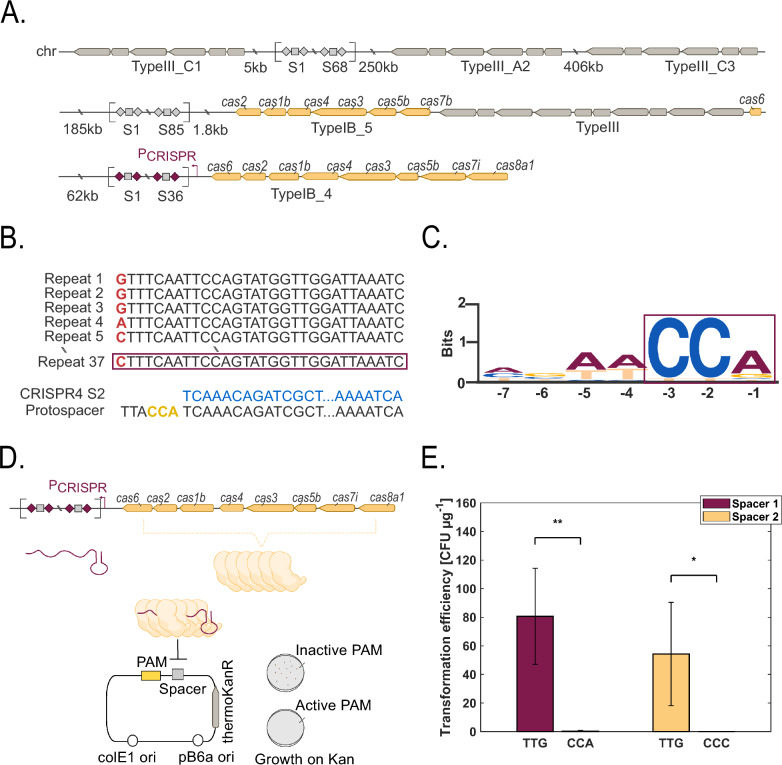
Investigation of the functionality of a type I-B CRISPR locus in *T. kivui.* **A** Genetic architecture of the CRISPR systems found in *T. kivui*. Squares represent spacer sequences; diamonds represent repeat regions. Spacer and repeat sequences are provided in Additional File 1, Table S1. **B** Sequence alignment of repeat regions from type I-B_4 CRISPR cluster of *T. kivui* identified with CRISPRCasFinder [34]*.* Non-conserved bases are highlighted in red (bold). The selected repeat sequence is highlighted with a box. CRISPRTarget [38] was used to find native protospacer targets in MGE databases. A representative example is shown, CRISPR4 S2 (spacer 2 from Type I-B_4 in blue, putative PAM sequence in yellow). **C** Putative PAM motif based on protospacer matches in CRISPRTarget [38]. Multiple sequence alignment of the reverse complement 3’ flank of protospacers visualized with Weblogo [53]). Source data are provided in Additional File 1, Table S2. **D** Plasmid interference assay design. The putative PAMs are inserted in a plasmid upstream of a spacer identified in *T. kivui* CRISPR cluster, and functional PAM sequences are determined by antibiotic challenge. **E** Results from plasmid interference assay. Data represent three biological replicates (average ± standard deviation)

To identify potential native CRISPR clusters and Cas genes on the genome of wild-type *T. kivui* (DSM 2030, GCA_000763575.1) we used the CRISPRCasFinder tool [34]. The results indicate that the genome of *T. kivui* encompasses three CRISPR arrays, as shown before [35], and five Cas systems—three type III and two type I-B (Fig. 1A, Additional File 1, Table S1). The Type III system was excluded, due to the requirement of active transcription of the targeted gene, which might limit the method to highly expressed genes [36]. The second type I-B CRISPR array of *T. kivui* (position: 2,344,710–2,347,139) was chosen for this study due to better targets found later in mobile genetic element (MGE) databases, compared to the largest type I-B array (position: 2,268,103–2,273,799) (Fig. 1A, Additional File 1, Table S1) [37]. In the selected CRISPR array, the native direct repeats have a well-conserved 30-nt sequence, and the 36 spacers have an average length of 36.6 bp (Fig. 1B, Additional File 1, Table S1). The most conserved direct repeat (5’-CTTTCAATTCCAGTATGGTTGGATTAAATC-3’) and spacers with a size of 36–37 bp were subsequently used (Fig. 1B, Additional File 1, Table S1). For efficient recognition of the protospacers by the Cas proteins, a functional PAM is required, which in type I-B systems is typically 3–5 bp long at the 5’ end of the protospacer (in the targeted DNA) [25, 26, 32]. Because the PAM sequence can only be found in the DNA initially targeted by the CRISPR system (i.e., not on *T. kivui* chromosome), we aligned the spacers found in *T. kivui* with MGE databases to identify functional PAM motifs. To that end, we used the CRISPRTarget tool [38], which showed the top targets to bear a CCN (N = any nucleotide) motif on the 5’ end of the protospacer (Fig. 1C, Additional File 1, Table S2). To verify that prediction, we designed a plasmid interference assay using CCA as the best match, and CCC as a secondary match (Fig. 1D). The resulting motif (CCM) can be found at least once in each coding sequence (CDS) of *T. kivui* and on average every 25.5 bp throughout the genome. Thus, editing of any given region of the genome (e. g. for gene knock-in) is possible as the probability of finding at least one PAM in a random stretch of DNA of 100 and 500 bp length is 98 and 100%, respectively.

For the plasmid interference assay, three different 37 bp spacers were introduced in a replicative vector with the chosen PAMs (CCA, CCC) cloned upstream of the protospacer. A potentially inactive PAM (TTG) was included as a control. *T. kivui* was transformed with the corresponding plasmids, and no colonies were obtained with both the CCA and CCC PAM-containing plasmid, compared to ~ 80 ± 34 CFU μg^−1^ for the plasmid which contained the TTG PAM (Fig. 1E). These results indicate that the spacer present on the plasmid is recognized as foreign sequence (protospacer) when a functional PAM is present (CCA, CCC), which triggers a DSB, eliminating the plasmid. Overall, the experiment demonstrates that the selected CRISPR type I-B system is active in* T. kivui.*

### Improvement of transformation efficiency in *Thermoanaerobacter kivui*

Due to the low transformation efficiency observed (80 ± 34 CFU μg^−1^), we decided to optimize the transformation protocol. Plating efficiency is relatively low in *T. kivui*, and we therefore first attempted to circumvent this problem. To that end, we switched the solidification agent to Gelrite (gellan gum), which is a common choice in thermophiles because of its higher melting temperature. Gelrite has been suggested to outperform agar for plating some microbes as contaminating compounds found in agar might inhibit bacterial growth [39]. Additionally, we reduced the plating volume to decrease the local solidification effect, and transformed and plated at 55 °C instead of 66 °C, based on previous experiments with the fluorescent reporter system FAST suggesting, that the plasmid is unstable at higher temperatures. While at 66 °C the transformation was completely abolished (0 CFU μg^−1^), we were able to increase the transformation efficiency by ~ 245-fold (1.96 × 10^4^ ± 8.7 10^3^ CFU μg^−1^) at 55 °C compared to the original protocol at 66 °C (80 ± 34 CFU μg^−1^). All transformations were subsequently performed using the improved protocol.

### Genome targeting with *T. kivui* type I-B CRISPR system

**Fig. 2:**
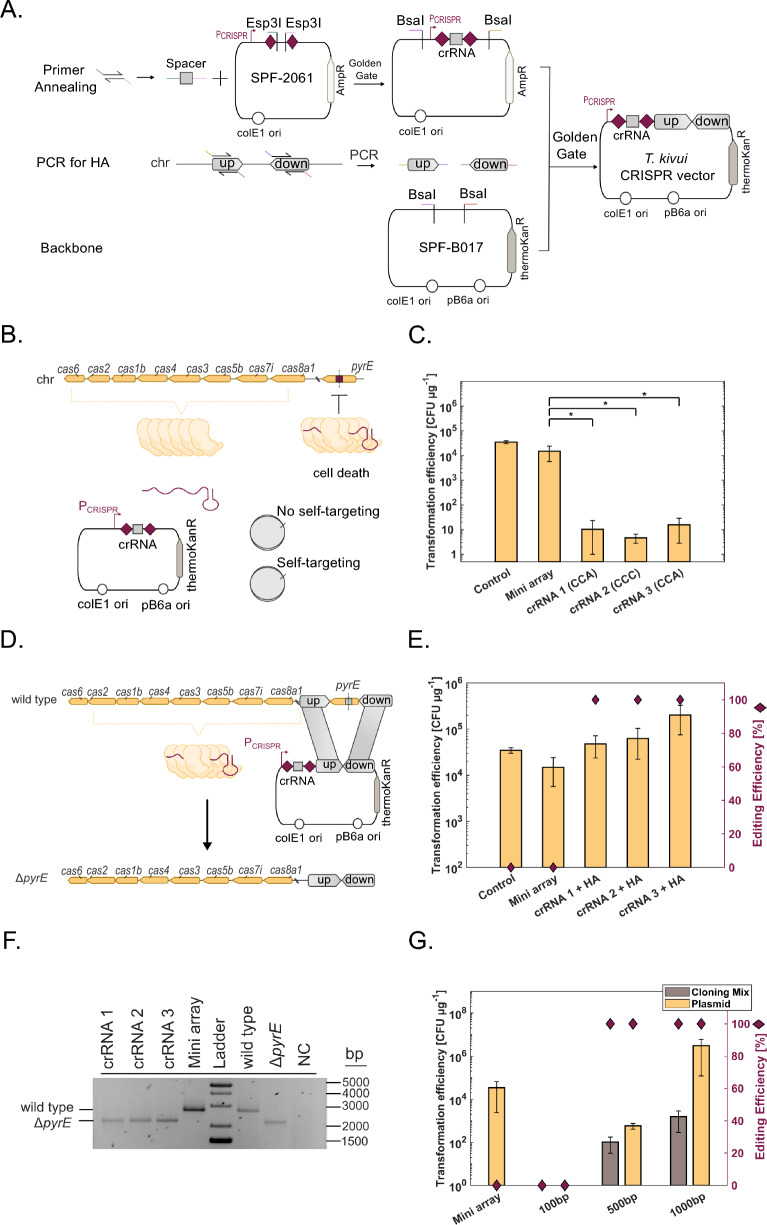
Development of a proof-of-concept for genome editing in *T. kivui* with its endogenous type I-B CRISPR system. **A** Cloning design with hierarchical Golden Gate assembly to generate the CRISPR plasmids. **B** Genome interference assay design. A plasmid bearing a synthetic mini array with crRNAs targeting the *pyrE* gene is transformed in *T. kivui*. Functionality of the CRISPR system is determined by antibiotic challenge. **C** Genome interference assay shows strain transformation is impaired for plasmids with *pyrE* crRNAs (CCA or CCC PAM). Controls: empty vector, mini array without cRNA. Data represent three biological replicates (average ± standard deviation). Statistical significance was determined using a two-tailed t-test for independent samples (*p < 0.05). **D** Design of CRISPR-based *pyrE* deletion. The functionality of the CRISPR system is determined by antibiotic challenge and PCR. **E** Transformation efficiencies of type I-B CRISPR vectors and editing efficiency of *pyrE* deletion. Controls: empty vector, mini array without crRNA. Transformation efficiency was measured for four independent experiments (average ± standard deviation), and editing efficiency is shown as the fraction of edited colonies (ten colonies, average ± standard deviation). **F** Gel electrophoresis of PCR results from (**E**). Data are representative of ten colonies per sample. **G** Effect of homology arm length and plasmid nature (purified from *E. coli* or Golden Gate assembly mixture) on *pyrE* deletion. Control: mini array without crRNA plasmid. Transformation efficiency was measured for four independent experiments (average ± standard deviation), and editing efficiency is shown as the fraction of edited colonies (ten colonies, average ± standard deviation)

Next, we tested the ability of the CRISPR method to target the genome of *T. kivui*. For this purpose, three genome-targeting vectors were constructed with an artificial mini array containing the native CRISPR array promoter and three crRNAs targeting the *pyrE* gene (Fig. 2A). Upon transformation of the resulting plasmids in *T. kivui* (Fig. 2B), a drastic decrease in transformation efficiency to 0.01% of the control was observed, demonstrating that the endogenous type I-B CRISPR system could be leveraged to target the genome of *T. kivui* (thereby preventing transformation, Fig. 2C). Although some colonies seemingly evaded the system, these were not able to grow again on medium supplemented with kanamycin.

Addition of a repair template (homology arms upstream and downstream of the *pyrE* gene) to the plasmid containing the crRNA provides the cell with an escape mechanism involving HR, leading to deletion of both the *pyrE* gene and cognate crRNA target sequence (Fig. 2D). In that configuration, a transformation efficiency of 4.7 × 10^4^ ± 2.4 × 10^4^ CFU μg^−1^, 6.2 × 10^4^ ± 4 × 10^4^ CFU μg^−1^ and 2 × 10^5^ ± 1.2 × 10^5^ CFU μg^−1^ was obtained for crRNA 1, 2 and 3, respectively (Fig. 2E). Colony PCR was performed for ten colonies per construct to identify *pyrE* deletion and demonstrated 100% editing efficiency for all three crRNAs, indicating a very high HR efficiency (Fig. 2F). Interestingly, the transformation efficiency increased for the vectors targeting the *pyrE* gene, compared to the empty vector containing no HAs and crRNA.

### CRISPR genome editing with shorter homology arms and Golden Gate-assembled plasmid

Type I-B CRISPR-based genome editing in *T. kivui* is 100% efficient. However, plasmid construction in *E. coli* frequently proved laborious and occasionally even impossible, especially with the addition of the homology arms (HAs) to the editing vector (often > 10 rounds of cloning). Therefore, we decided to reduce the size of the HAs from 1000 bp (demonstrated above) to 500 or 100 bp, which strongly facilitated cloning in *E. coli*. Additionally, short HAs come with lower DNA synthesis costs, thus circumventing some or all cloning steps, if desired. The plasmids with 1000-bp and 500-bp HAs were transformed in *T. kivui* and a transformation efficiency of 4 × 10^6^ ± 2 × 10^6^ CFU μg^−1^ and 5 × 10^2^ ± 1 × 10^2^ CFU μg^−1^ was observed, respectively (Fig. 2G). Colony PCR on 10 clones per construct was used to verify the *pyrE* deletion for these strains, demonstrating that despite the drastic drop in transformation efficiency, editing is still possible from 500-bp HAs and 100% efficient (Additional File 2, Fig. S1). The plasmids with 100-bp HAs did not result in any transformants, indicating that lower threshold for HA length is between 100 and 500 bp.

We further decided to test whether the *E. coli* cloning steps could be completely circumvented. To that end, the Golden Gate assembly cloning mixture, containing four parts (crRNA, upstream HA, downstream HA and the vector backbone (SPF-B017), Fig. 2A), was directly introduced in *T. kivui*. Genome editing was successful for the constructs with 1000 and 500 bp HA (1.5 × 10^3 ^± 1.2 × 10^3 CFU^ μg^−1^, 1 × 10^2^ ± 7.1 × 10 CFU μg^−1^, respectively), corresponding to a 2,555– and 5-fold decrease compared to plasmid DNA (Fig. 2G). Similar to the plasmid transformation results, the editing was 100% efficient for 500 and 1000 bp HAs but proved inefficient for 100 bp HAs (Additional File 2, Fig. S1). Collectively, the CRISPR-based genome editing tool can even be used directly with DNA assembled in vitro via Golden Gate cloning without the need for *E. coli* as a cloning host, thus enabling rapid genome editing.

### *rexA* negatively regulates the lactate dehydrogenase expression

The developed genome editing tool was used to characterize the function of the transcriptional regulators *rexA* (redox-sensing, TKV_RS02645) and *hrcA* (temperature-sensing, TKV_RS04760) in *T. kivui*. HrcA is a transcriptional repressor that regulates heat shock genes by binding to the CIRCE element, thereby playing a critical role in the organism’s response to heat stress [40]. Deletion of *hrcA* might de-repress the heat shock genes, which could improve temperature tolerance and promote an improved growth phenotype at higher temperatures. However, a *hrcA* deletion mutant did not show a significant phenotypic change during growth at 72 °C (data not shown), the maximum growth temperature of *T. kivui* [10].

**Fig. 3:**
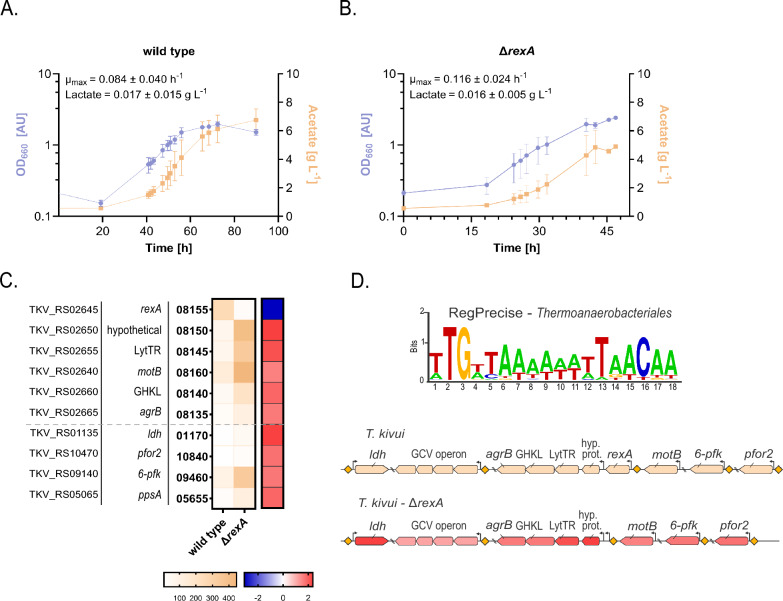
Characterization of the Δ*rexA* mutant in heterotrophic batch fermentation. **A**, **B** Growth and product formation of wild type (**A**) and Δ*rexA* (**B**) in batch fermentation with excess glucose (50 g L^−1^). Data represent three biological replicates (average ± standard deviation). The maximum specific growth rate µ_max_ was calculated from time point 43 and 53 for the wild type and from time point 31 and 40 for Δ*rexA*. **C** Results of RNA-seq analysis are shown as heatmap of the ten most differentially expressed genes in Δ*rexA* compared to the wild type. Log_2_ fold change differences (> ± 1.5-fold) between wild type and Δ*rexA* (blue: downregulation, red: upregulation) as well as TPM values from triplicates are shown. Locus tags are from the G-1 reference genome (OZ020628) [9]. Genes without formal gene name are displayed per the first word of the product name (LytTR = LytTR family DNA-binding domain-containing protein, GHLK = GHKL domain-containing protein). A grey line separates the genes found in the *rexA* locus from those involved in sugar metabolism. **D** Top: Sequence logo generated with the consensus sequence for *Thermoanaerobacterales* from the RegPrecise database [54]. Bottom: Schematic representation of the genetic architecture of a subset of differentially expressed genes in *T. kivui* and *T. kivui* Δ*rexA*. Colors are taken from (**C**) for log2-fold expression. Yellow diamond: Rex recognition motif. Arrow: Promoter. Source data can be found at Additional File 1, Table S4 and Table S5

The redox-sensitive transcriptional regulator RexA plays a major role in regulating gene expression in bacteria, in response to the intracellular redox state, particularly the NADH/NAD^+^ ratio [41]. In high NADH/NAD^+^ ratios, RexA is released from the DNA-binding site on the promoter region (Rex recognition site), leading to activation of the target genes involved in fermentation (e.g., alcohol dehydrogenase ADH, or lactate dehydrogenase LDH), H_2_ production and NAD biosynthesis [25, 42, 43]. The *rexA* deletion mutant was created by transforming plasmid SPF-3043, bearing a crRNA found in *rexA* and 1000 bp HAs. Heterotrophic batch cultures of the wild-type and Δ*rexA* strains were performed with a high glucose concentration (50 g L^−1^). Excess substrate can lead to overflow metabolism and therefore a high NADH/NAD^+^ ratio, which in anaerobic bacteria can be regenerated by production of lactate (via LDH) or ethanol (not produced by *T. kivui*) [11, 41, 43]. No significant difference was observed between the Δ*rexA* and wild-type strain in growth rate (0.116 ± 0.024 vs. 0.084 ± 0.040 h^−1^, respectively), as well as for acetate and lactate formation (Fig. 3A, B). Transcriptomics data analysis showed *ldh* (TKV_RS01135) to be the 5th most differentially expressed gene in the Δ*rexA* strain with a twofold upregulation (log_2_(FC) = 2.34) compared to the wild type (Fig. 3C, Additional File 1, Table S4). Genes found in the *rexA* locus were among the top most upregulated genes, including genes involved in sugar (*pfor2, ppsA, 6-pfk*) and glycine metabolism (*gcv*, glycine cleavage system) (Fig. 3C).

Rex regulates the expression of its target genes by binding to a Rex recognition sequence found in the promoter region [44]. Since RexA has not been studied in *T. kivui* before, we sought to identify the RexA recognition motifs. To that end, the consensus recognition sequence identified for two *Clostridia* groups (*Clostridiaceae* and *Thermoanaerobacterales*) (5’- T**TGTT**AANNNNT**TAACAA**-3’, bold represents highly conserved sites) was used as search query against the *T. kivui* genome (Fig. 3D). The presence of a RexA recognition site upstream of lactate dehydrogenase (*ldh*) has been described for some *Clostridia* [45]. In *T. kivui*, one Rex recognition site with an overall sequence identity of 83%, compared to the consensus sequence, was found upstream of the *ldh* gene (Additional File 1, Table S4). The fact that only minor amounts of lactate were formed during glucose fermentation indicates that CO_2_ fixation via the WLP is preferred over lactate formation for regeneration of reduction equivalents despite the upregulation of *ldh*. Collectively, these results indicate that RexA is a regulator of *ldh* in *T. kivui*.

Additionally, we identified a motif with 100% sequence similarity to the query at the promoter of the [FeFe] hydrogenase H-cluster radical SAM maturase HydG (TKV_RS05350), as previously shown for other *Thermoanaerobacterales* [41]. When allowing for up to 4 mismatches (2 at the highly conserved sites), the Rex recognition sequence was also found next to *pfor2* (pyruvate:ferredoxin oxidoreductase, TKV_RS10470), *6-pfk* (6-phosphofructokinase, TKV_RS09140) and *gcv* (glycine cleavage system operon (*gcvT, gcvH, gcvPA, gcvPB*), TKV_RS01195—TKV_RS01180), which were upregulated in the Δ*rexA* strain. Other genes for which the Rex recognition sequence was found include the hydrogenase maturation gene *hydE* (TKV_RS08520), the NAD^+^-dependent NADPH:Fd oxidoreductase Nfn (*nfnA, nfnb,* TKV_RS10870-75), a FAD-dependent oxidoreductase (TKV_RS09115) and a sulfurtransferase (from the TusA family, TKV_RS00425), which were not differentially expressed in this batch fermentation (Additional File 1, Table S4, Table S5). These results correlate with the rex regulons identified for *Clostridiaceae* and *Thermoanaerobacterales*.

### Curing of the vector following genome editing

During analysis of the transcriptomic data of Δ*rexA*, we detected reads corresponding to the kanamycin resistance gene, although the strain had undergone an initial curing procedure, after which the strain was unable to grow in the presence of kanamycin. Therefore, we developed a more rigorous curing protocol to ensure clean edited strains devoid of selection markers can be reliably obtained, thus enabling subsequent multiple genome editing rounds—a feature highly desirable to generate industrially-relevant strains.

To facilitate efficient curing of the edited strains, we first limited the use of antibiotics to the plating level. Curing of the transformed clones was performed by cultivating the clones at 66 °C. We hypothesized, based on the transformation efficiency results and previous findings [16], that the plasmid is unstable at 66 °C. Isolation with and without antibiotic selection was performed to identify the cured clones and estimate the curing efficiency. Screening for plasmid-free strains was done in liquid cultures in multi-well plates. As a proof-of-concept, the Δ*pyrE* strain was used. After three subcultures in liquid culture (~ 15 generations) and plating, 50% of the clones carrying the mini array plasmid were cured (n = 10, SPF-3040), whereas 60% were cured for the plasmid targeting the *pyrE* gene with 1000-bp HAs (n = 10, SPF-3037) (Additional File 2, Fig. S2). When we sought to cure the strains that were transformed with the 500-bp HA plasmids, we observed 100% curing efficiency after 2 subcultures in liquid culture (~ 9 generations, n = 10, SPF-3078) (Additional File 2, Fig. S2). These results demonstrate that shortening the HAs drastically reduces the time required to acquire a cured, clean strain.

### Generation of a single nucleotide mutation in *T. kivui*

The introduction of single nucleotide polymorphisms (SNP) is usually harder to achieve with low gene editing efficiencies. Recently, a point mutation was introduced with 6% editing efficiency in the gene coding for the PolC-type DNA polymerase III from *A. thermocellus*, resulting in a hypermutator phenotype displaying a 30–178-fold higher mutation rate compared to the wild type [46]. Therefore, we chose the same mutation (targeting the corresponding conserved residue in *T. kivui*, TKV_RS06370) to demonstrate the efficiency of Hi-TARGET in creating point mutations, but also to potentially accelerate adaptive laboratory evolution (ALE) experiments [47].

Using Hi-TARGET, three point mutations were introduced in *T. kivui* by transforming plasmid SPF-4052, containing a crRNA targeting *polC* and an HA containing the target point mutation and two silent mutations (to provide an HR template not targeted by the CRISPR system). The cells were cured after 5 serial transfers with an inoculum size of 0.1% (v/v) over 4 days. The mutation rate was calculated after conducting a mutation accumulation transfer in H_2_/CO_2_ for ~ 140 generations. The calculated mutation rate per generation of the PolC^C629Y^ was determined to be 0.047 ± 0.015 generation^−1^, which is ~ 60% lower compared to the wild type (0.132 ± 0.007 generation^−1^, Additional File 2, Fig. S3, Additional File 1, Table S6). Although no hypermutator was created, we demonstrated the ability of Hi-TARGET to precisely edit single nucleotides with an editing efficiency of 49%, significantly higher compared to the editing efficiency obtained with other thermoanaerobes (e.g. 6% editing efficiency in *A. thermocellus*) [46].

### Integration of a fluorescent marker (pFAST) in *T. kivui*

**Fig. 4:**
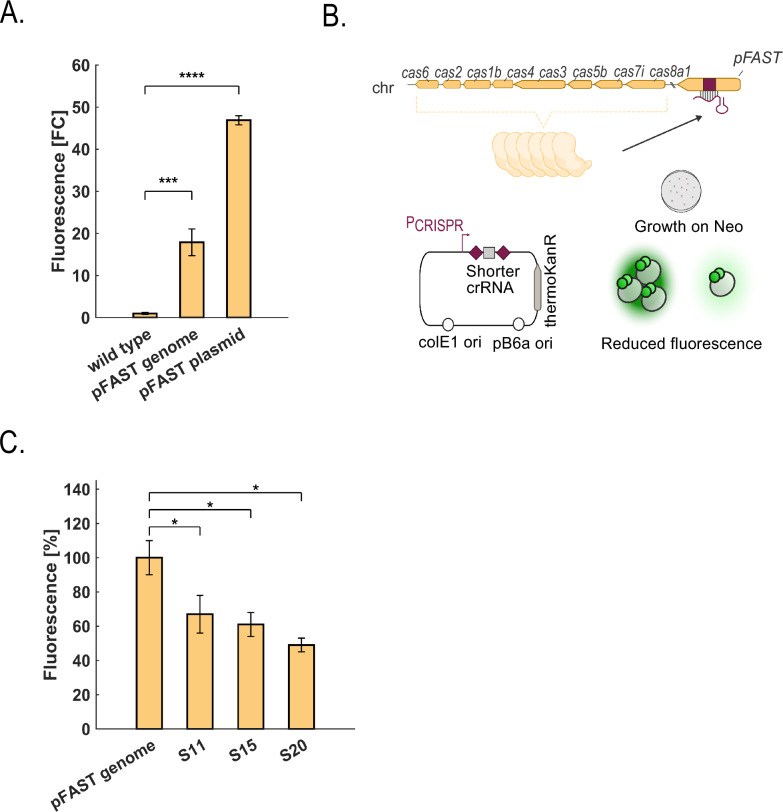
Knock-down of the integrated fluorescent protein pFAST in *T. kivui*. **A** Fluorescence quantification of *T. kivui* cells expressing pFAST from a plasmid or the genome. Data represent three biological replicates (average ± standard deviation). **B** Design of CRISPR-based knock-down of *pFAST*. Plasmids expressing short crRNAs are introduced in *T. kivui*. The short crRNA interferes with *pFAST* transcription, which should result in reduced fluorescence. **C** Results of fluorescence measurements obtained with short spacers in the crRNA (S11: 11 bp, S15: 15 bp, S20: 20 bp) targeting *pFAST* in *T. kivui*. Data represent 5–7 biological replicates and are displayed as percentage average ± standard error. Statistical significance was determined using a two-tailed t-test for independent samples (*p < 0.05, ***p < 0.001, ****p < 0.0001)

The developed tool was used to demonstrate whether a gene knock-in would also be possible in *T. kivui*. To that end, we chose to integrate the fluorescent reporter *pFAST* gene at the locus of the lactate dehydrogenase (*ldh*). The integration of the marker could potentially alleviate plasmid-related instability that previously seemed to affect FAST-mediated fluorescence at 66 °C, the optimal growth temperature of *T. kivui* [16]. Following successful integration of *pFAST* into the genome, the edited strain displayed a ~ 20-fold higher fluorescence compared to the wild type, which corresponds to 42% of the fluorescence observed for plasmid-based expression of *pFAST* (Fig. 4A). Similar to our previous findings, fluorescence was only detected at 55 °C and not at 66 °C (Additional File 2, Fig. S4) [16].

### Shorter crRNA enables gene knockdown of pFAST in *T. kivui*

An efficient knock-down system is important in cases where gene deletion is not possible. Previously, gene knock-down was demonstrated in *T. kivui* with the use of sugar-inducible promoters, which can be limited for other substrates (e.g., CO, H_2_/CO_2_) [14]. In *P. thermoglucosidasius*, gene knock-down was observed when shorter crRNA were used to approximately 85% [32]. To test the efficiency of gene knock-down with Hi-TARGET in *T. kivui*, the *pFAST* strain was cured using the procedure described above. The fluorescent signal obtained in strains with crRNAs of different lengths targeting the *pFAST* gene served as a proxy for gene knock-down (Fig. 4B). Mini array plasmids were designed to contain spacers targeting the *pFAST* coding sequence at a length of 11, 15, and 20 instead of 36 bp (the canonical crRNA length) (Table 1, Additional File 1, Table S3). The plasmids were transformed in *T. kivui* with transformation efficiency of 8.7 × 10^2^ ± 5.5 × 10^2^ CFU μg^−1^, 4.4 × 10^2^ ± 3.8 × 10^2^ CFU μg^−1^ and 3.1 × 10^3^ ± 1.2 × 10^3^ CFU μg^−1^ for spacer 11, 15 and 20, respectively, compared to the control (2.8 × 10^3^ ± 1.1 × 10^3^ CFU μg^−1^) (Additional File 2, Fig. S5). Next, fluorescence quantification demonstrated significant reduction in correlation to the spacer length, with a maximum KD of 52% (Fig. 4C). Colony PCR and Sanger sequencing on the *ldh*::*pFAST* strain with spacer 20 confirmed that the plasmid and the genome with the *pFAST* coding sequence remained intact and the reduced fluorescence was not due to inefficient Cas activity. These results indicate that CRISPRi could drive gene knock-down in *T. kivui*. Higher downregulation levels could potentially be achieved by targeting the promoter region as demonstrated in *H. hispanica* [33] and using spacers longer than 20 bp [32].

**Table 1:** Strains used in this study

*E. coli* DH10B	Cloning strain	Thermo scientific
Strains		
*T. kivui* DSM 2030	Acetogen, T_opt_ = 66 °C	DSMZ
TKV_0020	Derivative of DSM 2030	This work
ASI_TKV_0001	TKV_0020, SPF-B017	This work
ASI_TKV_0005	TKV_0020, SPF-3037 (Δ*pyrE*)	This work
ASI_TKV_0006	TKV_0020, SPF-3038 (Δ*pyrE*)	This work
ASI_TKV_0007	TKV_0020, SPF-3039 (Δ*pyrE*)	This work
ASI_TKV_0008	TKV_0020, SPF-3040 (mini array)	This work
ASI_TKV_0019	DSM 2030, GGA with SPF-2070, 500 bp HAs (PCR SPF-3039 ASI229-230), SPF-B017 (Δ*pyrE*)	This work
ASI_TKV_0020	DSM 2030, DSM 2030, GGA with SPF-2070, 1000 bp HAs (PCR SPF-3039 ASI132-133), SPF-B017 (Δ*pyrE*)	This work
ASI_TKV_0021	DSM 2030, SPF-3078 (Δ*pyrE*)	This work
ASI_TKV_0029	DSM 2030, SPF-3047 (Δ*rexA*)	This work
ASI_TKV_0031	DSM 2030, SPF-3084 (Δ*hrcA*)	This work
ASI_TKV_0032	DSM 2030 SPF-3063 (*ldh*::pFAST)	This work
ASI_TKV_0033	DSM 2030 SPF-3064 (*ldh*::pFAST)	This work
ASI_TKV_0041	DSM 2030 SPF- B017	This work
ASI_TKV_0050	DSM 2030 Δ*hrcA*, cured colony	This work
ASI_TKV_0051	DSM 2030, *ldh*::pFAST, cured colony	This work
ASI_TKV_0052	ASI_TKV_0051, SPF-3093 (*ldh*::pFAST, spacer 20)	This work
ASI_TKV_0053	ASI_TKV_0051, SPF-3094 (*ldh*::pFAST, spacer 15)	This work
ASI_TKV_0054	ASI_TKV_0051, SPF-3095 (*ldh*::pFAST, spacer 11)	This work
RHO_TKV_0010	TKV_0020, SPF-2015 (pThermoFAST_01)	[16]
ALU_TKV_0009	DSM 2030, SPF-4052 (PolC^C629Y^)	This work
*E. coli—Thermoanaerobacter* shuttle plasmids		
SPF-B009	pMU131 engineered for Bbs1 GGA cloning, Recipient Sites 1–4, removable amilCP	[16, 55]
SPF-2021	SPF-B009, active PAM and Spacer 1 (RH050-051)	This work
SPF-2022	SPF-B009, inactive PAM and Spacer 1 (RH052-053)	This work
SPF-2023	SPF-B009, active PAM and Spacer 2 (RH054-055)	This work
SPF-2024	SPF-B009, inactive PAM and Spacer 2 (RH056-057)	This work
SPF-B017	B6A-RI ori engineered for Bsa1 GGA cloning, recipient Sites A-D, colE1 origin of replication, thermoKanR	This work
SPF-2061	Synthetic mini array containing direct repeats, Esp3I recipient sites 2,3, colE1, ampR	This work
SPF-2068	SPF-2061, crRNA 1 (RH058-059) for *pyrE*	This work
SPF-2069	SPF-2061, crRNA 2 (RH060-061) for *pyrE*	This work
SPF-2070	SPF-2061, crRNA 3 (RH062-063) for *pyrE*	This work
SPF-3037	SPF-2068, 1000 bp HAs (ASI132-133), SPF-B017	This work
SPF-3038	SPF-2069, 1000 bp HAs (ASI132-133), SPF-B017	This work
SPF-3039	SPF-2070, 1000 bp HAs (ASI132-133), SPF-B017	This work
SPF-3040	SPF-2061 (AD PCR, ASI168-169), SPF-B017	This work
SPF-3041	SPF-2068 (AD PCR, ASI168-169), SPF-B017	This work
SPF-3042	SPF-2069 (AD PCR, ASI168-169), SPF-B017	This work
SPF-3043	SPF-2070 (AD PCR, ASI168-169), SPF-B017	This work
SPF-3077	SPF-2070, 100 bp HAs (PCR SPF-3039 ASI231-232), SPF-B017	This work
SPF-3078	SPF-2070, 500 bp HAs (PCR SPF-3039 ASI229-230), SPF-B017	This work
SPF-2094	SPF-2061, crRNA *hrcA* (ASI145-146)	This work
pMini2.0	PCR cloning kit vector	NEB
SPF-2157	*hrcA* right HA (1000 bp, ASI151-152), pMini2.0	This work
SPF-2158	*hrcA* left HA (1000 bp, ASI153-154), pMini2.0	This work
SPF-3084	SPF-2094, SPF-2157, SPF-2158, SPF-B017	This work
SPF-2093	SPF-2061, crRNA for *rexA* (ASI160-161)	This work
SPF-3047	SPF-2093, *rexA* HAs (1000 bp, ASI164-165, ASI166-167), SPF-B017	This work
SPF-2119	SPF-2061, crRNA 1 for *ldh* (ASI195-196)	This work
SPF-2120	SPF-2061, crRNA 2 for *ldh* (ASI197-198)	This work
SPF-2159	*ldh* right HA (1000 bp, ASI199-200), pMini2.0	This work
SPF-2015	SPF-B009, Expression of pFAST with Ppta, tKan	[16]
SPF-2160	*ldh* left HA (1000 bp, ASI201-202), pMini2.0	This work
SPF-3063	SPF-2119, SPF-2159, PCR XY SPF-2015 (ASI203-204), SPF-2160, SPF-B017	This work
SPF-3064	SPF-2120, SPF-2159, PCR XY SPF-2015 (ASI203-204), SPF-2160, SPF-B017	This work
SPF-3093	crRNA (20 bp) for *pFAST* (ASI240-241), SPF-B017	This work
SPF-3094	crRNA (15 bp) for *pFAST* (ASI242-243), SPF-B017	This work
SPF-3095	crRNA (11 bp) for *pFAST* (ASI244-245), SPF-B017	This work
SPF-B030	Same as SPF-B017 with the Pthl promoter (C*. acetobutylicum)* for thermoKanR	This work
SPF-4051	pTwist, AmpR, crRNA for *polC*, *polC* HA (C669Y), fusion sites AD	Synthesis
SPF-4052	SPF-4051, SPF-B030	This work

## Conclusion

This work presents the efficient, versatile genome editing method Hi-TARGET for *T. kivui* engineering based on its endogenous type I-B CRISPR system. We identified key genetic elements (PAM, spacer length, CRISPR array promoter, direct repeats) required for *T. kivui* genome editing and demonstrated high efficiency with different sizes of HAs.

The Hi-TARGET method involves first a two-step cloning procedure: assembling crRNA and HAs elements via Golden Gate assembly in a shuttle vector (SPF-B017). At this stage, there are two options: the Golden Gate reaction can be directly transformed into *T. kivui* (day 0), or the shuttle vector can be first transformed in *E. coli* (day 0–5 for 1000 bp HAs, day 0–3 for 500 bp HAs) prior to transformation into *T. kivui*.

Furthermore, we describe an optimized transformation protocol with increased transformation efficiency by 245-fold, enabling 100% edited clones (for knock-out and knock-in) within 5 days (day 0–5). A robust plasmid curing protocol was also developed, with complete loss achieved in ~ 9 (day 5–12 for 500 bp HAs) to ~ 22 (day 5–14 for 1000 bp HAs) generations. Overall, Hi-TARGET is able to generate clean, edited strains in as little as 12 days (cloning mix-based, 500 bp HAs) or 15 days (plasmid-based, 500 bp HAs).

Compared to other acetogens and thermophiles, the rapid growth of *T. kivui* significantly accelerates the process. In *A. woodii* transformation and plating alone require 10 days, compared to 5 days for *T. kivui* [29]. Genome editing in *T. kivui* based on Hi-TARGET is relatively fast even when compared to the fast-growing model organism *E. coli*, where genome editing with curing requires 7–8 days [48].

An important aspect of CRISPR-based genome editing are off-target effects. In *Clostridium tyrobutyricum*, the use of shorter spacers (< 20 bp) for genome editing led to cell death, possibly due to non-specific crRNA binding and an inability to repair DSBs [28]. With Hi-TARGET, successful gene KD was achieved using spacers as short as 11 bp without clear indications of off-target effects leading to cell death. In case of mistargeting by the crRNA, a repair template or an active non-homologous end joining (NHEJ) system may rescue the cells with acquired mutations. Since our genome interference assays (no repair template) in *T. kivui* did not yield any colonies, this hints that *T. kivui* appears to lack an efficient NHEJ (despite the presence of homologous genes). Therefore, unintended DSBs, if they occur, may be more likely to result in cell death rather than the accumulation of mutations, though further investigation is needed.

Hi-TARGET was validated for three gene deletions, point mutations, genetic integrations, and knockdowns, showcasing its versatility. This method enables rapid generation of clean, edited strains, offering the potential to advance both the understanding of acetogenic metabolism and contribute to industrial production of green chemicals and fuels.

## Materials and methods

### In silico identification of the endogenous type I-B CRISPR components of *T. kivui*

The database from CRISPRCasFinder [34] was used to find putative CRISPR loci in the *T. kivui* DSM 2030 genome. The second type I-B cluster was selected for further analysis (position: 2,344,710 to 2,355,164). Repeat sequence was used as predicted by CRISPRCasFinder. The putative spacers were extracted and used as a query in the CRISPRTarget program [38], using default parameters and a threshold score of 26. The putative PAM sequences were manually inferred from the complement of the sequences flanking the protospacer (Additional File 1, Table S2).

### Bacterial strains and culture conditions

All strains used in this study are described in Table 1.

*E. coli* DH10B was routinely cultivated in LB medium (10 g L^−1^ tryptone, 10 g L^−1^ NaCl, 5 g L^−1^ yeast extract) at 37 °C with carbenicillin (100 mg L^−1^) or kanamycin (50 mg L^−1^) as needed. For solid media, 15 g L^−1^ agar was added.

*T. kivui* was handled and maintained anaerobically as previously described [6]. All experiments were conducted in a vinyl anaerobic chamber (Coy lab, Grass Lake, United States) or in an anaerobic workstation (Concept 500, Baker Ruskinn, Bridgend, UK). The minimal medium (MM) used in this study is composed of (per liter): 5.5 g glucose monohydrate, 7.8 g Na_2_HPO_4_*2H_2_O, 6.9 g NaH_2_PO_4_*H_2_O, 210 mg K_2_HPO_4_, 160 mg KH_2_PO_4_, 250 mg NH_4_Cl, 225 mg (NH_4_)_2_SO_4_, 438 mg NaCl, 91 mg MgSO_4_*7H_2_O, 6 mg CaCl_2_*2H_2_O, 2 mg FeSO_4_*7H_2_O, 5.41 g KHCO_3_, 500 mg cysteine-HCl*H_2_O and 10 mL DSM141 modified Wolin’s mineral solution. Plates were prepared by adding 2 g L^−1^ yeast extract (MMY) and 3 g L^−1^ or 7.5 g L^−1^ (embedded or normal plating) Gelrite (Carl Roth GmbH & Co. KG, Karlsruhe, Germany) or 13 g L^−1^ or 8 g L^−1^ (embedded or normal plating) Bacto agar (BD, United States). 200 mg L^−1^ kanamycin or neomycin were added when required. Hungate tubes, 125 mL serum bottles sealed with rubber stoppers, 12- and 24-well plates were used for anaerobic cultivation. Prior to cultivation, the headspace was flushed with a gas mixture containing 80% N_2_ and 20% CO_2_ and the absolute pressure was set to 2 bar. An incubator (Infors Minitron, Infors HT, Bottmingen, Switzerland) or a shaking water bath (Memmert, Schwabach, Germany) were used for cultivation at 66 °C or 55 °C.

### Plasmid construction with Golden Gate assembly

*E. coli* DH10b was used as a cloning host. We followed a simplified hierarchical Golden Gate assembly system as demonstrated before [16, 49]. Briefly, DNA was amplified either from *T. kivui* genome or synthesized (Twist Biosciences, United States) with a compatible restriction enzyme (BsaI/Esp3I) and fusion site. The primers were ordered from Microsynth AG (Balgach, Switzerland) and are listed in Additional File 1, Table S3. An overview of the plasmids used in this study can be found in Table 1.

For the plasmid interference experiment, the native spacer with the 5’-flank sequence containing the predicted functional or inactive PAM (created with primer annealing of primers RH050-RH057, described in Additional File 1, Table S3) was assembled in a pMU131-based vector (SPF-B009, [16]).

To generate a CRISPR plasmid, we identified the functional PAM sequences in the target gene by searching for CCM motifs with CLC Workbench (Qiagen Inc., Hilden, Germany). We designed primers binding downstream of the active PAM with 36–37 bp length and overhangs corresponding to compatible fusion sites (2, 3). The primers were annealed in a thermocycler with 5 °C min^−1^ ramp down from 95 °C and assembled with Esp3I to a vector flanked by the repeat regions from *T. kivui* cluster Type I-B_4 (SPF-2061, A2, 3B), creating the crRNA plasmid. The shuttle *E.  coli*–*T. kivui* vector SPF-B017 was used as a backbone for all *T. kivui* constructs and was generated as described before [16].

For genome interference assays, the crRNA 1, 2, and 3 for *pyrE* was amplified from SPF-2061, SPF-2068, SPF-2069, SPF-2070 with primers ASI168-169 and assembled in SPF-B017.

The homology arms (100, 500 and 1,000 bp) were amplified with Phusion DNA polymerase (Thermo Fisher Scientific, Waltham, USA) with compatible fusion sites (B, C and C, D) and flanking BsaI sites and assembled with the crRNA plasmid and the SPF-B017 shuttle vector (A, D). For genomic insertion, new fusion sites (X: TGTC, Y: CCAA) were introduced between the upstream and downstream homology arms to be compatible with the pFAST expression cassette.

For the point mutation, the crRNA template was designed to target the mutation locus. The desired mutation was introduced in the HAs alongside two silent mutations in the crRNA to avoid recognition by the CRISPR system.

For the knock-down constructs, the spacers were designed downstream of a functional PAM as described above, but with a reduced length of 11, 15, and 20 bp. In this case, the spacers were directly introduced to the final vector SPF-3040 expressing the direct repeats without the presence of HAs.

### Plasmid/genome interference and genome editing

For PAM determination, 2 mL of competent *T. kivui* cells were transformed with the target plasmid DNA at concentrations of 1 μg mL^−1^ and allowed to recover by incubating at 66 °C overnight. To select the transformed cells, 2 mL of the cell suspension was plated under anoxic conditions into MMY with Bacto agar (8 g L^−1^) containing 5 g L^−1^ glucose and 200 mg L^−1^ kanamycin. The plates were then placed in a custom anaerobic jar (J. Fuchs GmbH, Hausbach, Austria), with 80% N_2_ and 20% CO_2_ and pressure set to 2 bar, at 66 °C until colony formation. Colony formation was counted after 3 days.

For genome interference and editing, the protocol was slightly altered to 0.5 mL of competent *T. kivui* cells transformed with the target DNA at concentrations of 1 μg mL^−1^ of plasmid or 50 μL of Golden Gate assembly mixture (~ 0.35 μg DNA) and grown at 55 °C overnight. To select the transformed cells, 0.1 mL of the cell suspension was plated under anoxic conditions into MMY with Gelrite (3 g L^−1^) containing 5 g L^−1^ glucose and 200 mg L^−1^ neomycin. The plates were then placed in a custom anaerobic jar, with 80% N_2_ and 20% CO_2_ and pressure set to 2 bar, at 55 °C until colony formation. The clones picked from the plates were incubated with MMYG and verified with colony PCR and Sanger sequencing (Microsynth, Balgach, Switzerland).

### Genotyping

PCR (B7 polymerase, Biozym, Hessisch Oldendorf, Germany) was performed with 0.5 μL of active culture. The primers (Additional File 1, Table S3) were designed to bind solely to the genome upstream and downstream of the target to avoid amplification of the plasmid and false-positive results.

### Plasmid curing

To remove the CRISPR plasmids from edited cells, the edited colony was inoculated into 2 mL of medium without kanamycin and cultivated at 66 °C for 16 h (sub-culture 1). The culture was serially transferred into fresh medium with an inoculum size of 0.1–0.01% (vol/vol) for two or three subcultures without antibiotics until a maximum OD_660_ was reached. The culture was streaked for colony isolation on plates containing MM, yeast extract (2 g L^−1^) and glucose (5 g L^−1^) at 66 °C for 2 days. Colonies were confirmed to have been cured by determining their sensitivity to kanamycin in liquid culture with well-plates.

### Batch fermentation

For batch fermentation of *T. kivui*, the mineral medium described above, excluding KHCO_3_, was used in 250 mL stirred-tank reactors (DASBox Mini Bioreactor system, Eppendorf AG, Hamburg, Germany) with a working volume of 200 mL at 150 rpm and 66 °C. An Easy Ferm Plus K8 120 pH electrode (Hamilton, NV, USA) was used for monitoring and pH adjustment with 5 M KOH pumped with a MP8 multi pump module (Eppendorf, AG, Hamburg, Germany). The gassing of the reactor (0.05 vvm) was controlled with internal mass flow controllers through a micro sparger (10 μm pore size, Sartorius Stedim Biotech GmbH, Göttingen, Germany). The reactor was frequently sampled to determine OD_660_ (ONDA V-10 Plus Visible Spectrophotometer) and product titer with HPLC (HPLC Ultimate 3000, Thermo Fisher Scientific, Waltham, MA, USA). The samples were analyzed using an Aminex HPX-87H column (300 × 7.8 mm, Bio-Rad, Hercules, CA, USA) at 0.6 mL min^−1^ of 4 mM H_2_SO_4_ for HPLC, equipped with a refractive index detector (Refractomax 520, Thermo Fisher Scientific, Waltham, MA, USA) and a diode array detector (Ultimate 3000, Thermo Fisher Scientific, Waltham, MA, USA) for quantification.

### RNA-seq

Cells at exponential phase (OD = 1) were harvested, pelleted and snap-frozen in liquid nitrogen. RNA-seq transcriptome library was prepared by Microsynth AG (Balgach, Switzerland) as previously described [9], with minor modifications. Briefly, cell pellets were resuspended using DNA/RNA Shield (Zymo Research, Irvine, CA, USA), and lysed through bead beating. RNA was extracted from the soluble fraction using the RNeasy Plus 96 Kit (Qiagen). Libraries were generated with the Stranded Total RNA Prep Kit with Ribo-Zero Plus (Illumina) according to the manufacturer's protocol, followed by sequencing on an Illumina NovaSeq 6000 system (2 × 150 bp). The resulting paired-end reads underwent demultiplexing and adaptor trimming using Illumina's bcl2fastq software (v2.20.0.422). For each sample, 4 million reads were randomly sampled using Seqkit (v2.4.0, [50]). Analysis of RNA-seq data was subsequently performed using Geneious Prime 2024.0.7 (http://www.geneious.com/). RNA-seq reads were trimmed using the Geneious built-in algorithm (error probability limit threshold 0.05) and aligned to the *T. kivui* wild-type genome (OZ020628) using the Minimap2 (v 2.24-r1122, [51]) plugin (default parameter, short read mode). Gene expression levels were quantified with Geneious (default parameters). Differential expression analysis was performed with the DESeq2 plugin [52] in Geneious, also using default parameters. Genes were classified as differentially expressed if |log2(fold change)|> 1 and the Benjamini–Hochberg adjusted p-value was < 0.05.

### ALE for mutation accumulation

Two biological replicates each for the wild type and PolC^C629Y^ were grown autotrophically (80% H_2_ and 20% CO_2_ at 2 bars, 66 °C) with an inoculum size of 5% (v/v) for 30 transfers (~ 140 generations). gDNA was extracted with the Quick-DNA Miniprep Plus Kit from Zymo Research (Irvine, CAL, USA). The gDNA from transfers 1, 10, 20, and 30 were used for PCR and Sanger sequencing to verify the PolC point mutation (PolC^C629Y^). The gDNA from transfer 1 and 30 were used for Illumina whole genome sequencing (Microsynth AG, Balgach, Switzerland).

### Whole genome sequencing and analysis

DNA libraries were prepared using the Illumina Tagmentation kit and sequenced with a NovaSeq 6000 sequencer (2 × 250 bp). The resulting paired-end reads underwent demultiplexing and adaptor trimming using Illumina's bcl2fastq software (v2.20.0.422). The data were analyzed with Geneious Prime 2024.0.7 (http://www.geneious.com/). Using BBDuk (v1.0), reads were trimmed for low quality (< Q20) and paired-end overhangs (minimum overlap of 20), and short reads (< 20 bp) were filtered out. The trimmed files were mapped to the reference *T. kivui* G1 wild-type genome (OZ020628) using the built-in Geneious mapper (default parameters). SNVs/indels were identified using the built-in function of Geneious, with a threshold for a minimal variant frequency of 35%. The synonymous variants in the transfer 30 DNA—but not present in transfer 1 DNA—were selected for mutation rate calculation. Mutation rate was determined by dividing the total number of synonymous SNPs in transfer 30 by the number of generations separating the transfer 1 and transfer 30 cultures.

### Fluorescence measurement

Fluorescence of pFAST was measured as described before [16]. Briefly, 5 OD units of cells grown without yeast extract and resazurin were harvested in exponential phase (OD ~ 1.5). The cells were resuspended in 70 mM sodium phosphate and 5 μΜ HMBR (Twinkle Factory, Paris, France) and placed in a 96-well plate (ref: 655209, Greiner, Kremsmünster, Austria). Fluorescence measurements were conducted in a plate reader (Infinite M200, Tecan, Männedorf, Switzerland) as described before. Results were normalized using the fluorescence value obtained for wild-type cells with buffer and without HMBR.

## Supplementary Information

Supplementary material 1.

Supplementary material 2.

## Data Availability

All data generated or analyzed during this study are included in this published article, Additional Files 1 and 2.
