# Supplementary material for: Hi-TARGET: a fast, efficient and versatile CRISPR type I-B genome editing tool for the thermophilic acetogen Thermoanaerobacter kivui
Source: Biotechnol Biofuels Bioprod. 2025 Apr 30;18:49. doi: 10.1186/s13068-025-02647-0 (PMC12044746; doi:10.1186/s13068-025-02647-0)
Supplement: Supplementary file 2 — Supplementary material 2. [file 13068_2025_2647_MOESM2_ESM.docx]

**Additional File 2**

Hi-TARGET: A fast, efficient and versatile CRISPR type I-B genome editing tool for the thermophilic acetogen *Thermoanaerobacter kivui*.


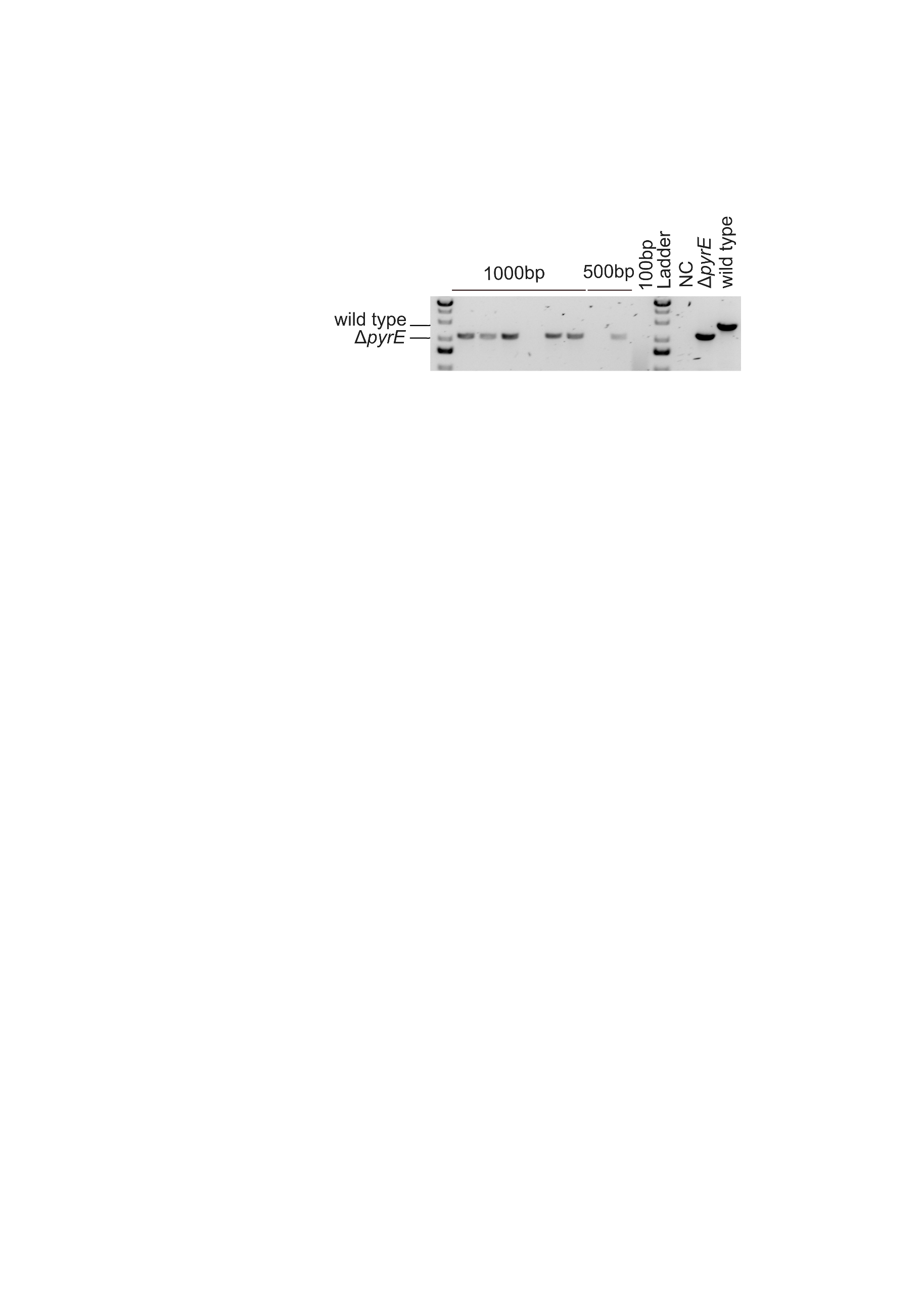


**Fig. S1. Editing efficiency of CRISPR with Golden gate mix.** PCR and gel electrophoresis targeting the *pyrE* locus. The localization of the wild type or Δ*pyrE* band is demonstrated on the left. The size of the homology arms is displayed on top. NC = negative control. Data are representative of 10 colonies per construct.


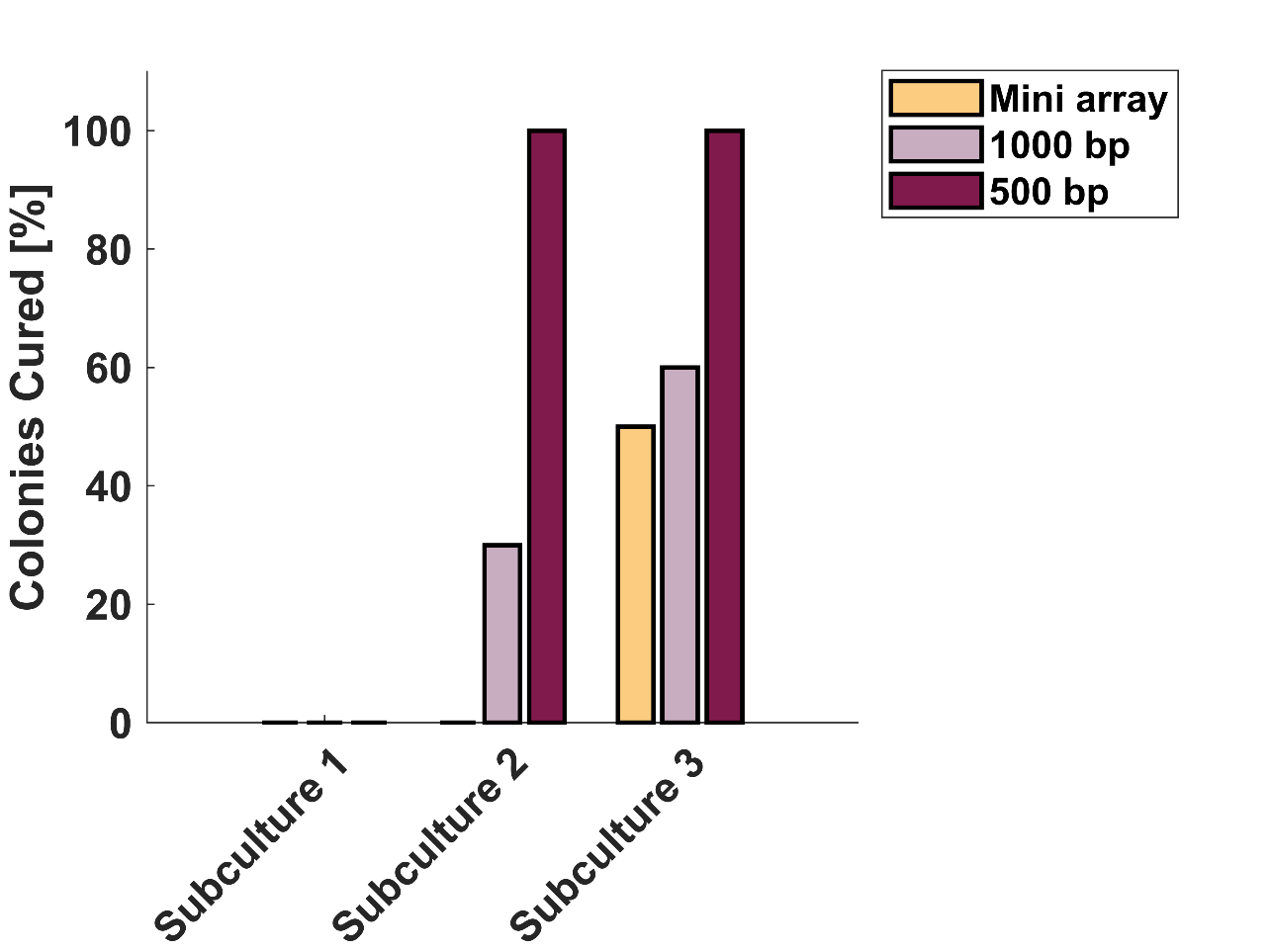


**Fig. S2 Curing efficiency.** The bars represent the fraction (%) of the colonies cured after 1, 2, or 3 subcultures. Clones were isolated and individually tested for growth on Kanamycin. Mini array: empty vector control, containing the direct repeats. 1000 bp: plasmid targeting the *pyrE* gene with 1000 bp HAs. 500 bp: plasmid targeting the *pyrE* gene with 500 bp of HAs. Colonies tested per sample: n=10.


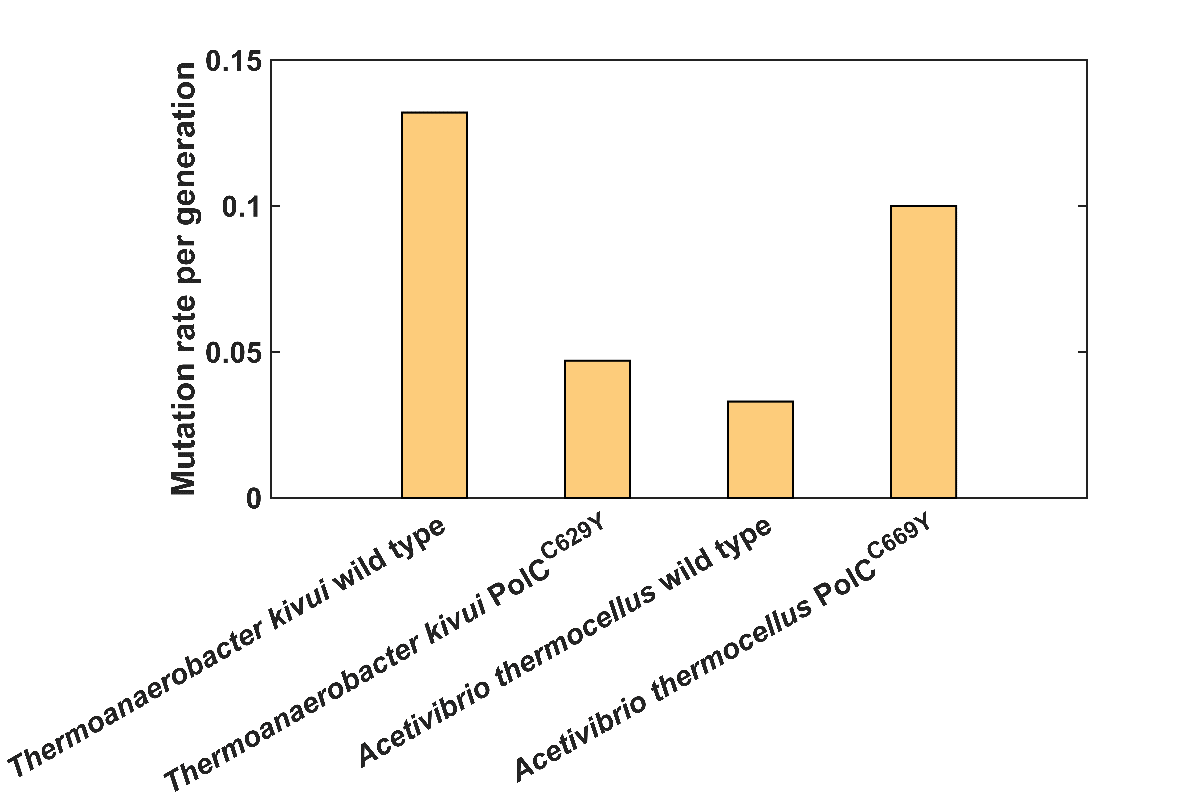


**Fig. S3 Mutation rate.** Comparison of the wild-type strain (wild type) mutation rate (based on synonymous mutations) and PolC mutant of *T. kivui* (PolC^C629Y^) and *C. thermocellum* (PolC^C669Y^). Direct comparison of the mutation rate between the organisms should be interpreted with caution due to variations in the methodological approach.


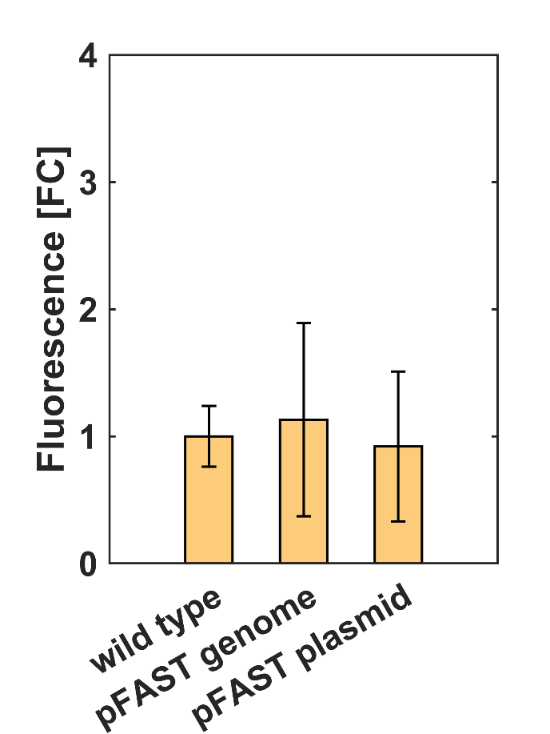


**Fig. S4 Fluorescence at 66 °C.** Log2-fold change difference of pFAST expressed from the genome or the plasmid compared to the wild-type strain, at 66 °C. Data represent three biological replicates (average ± standard deviation).


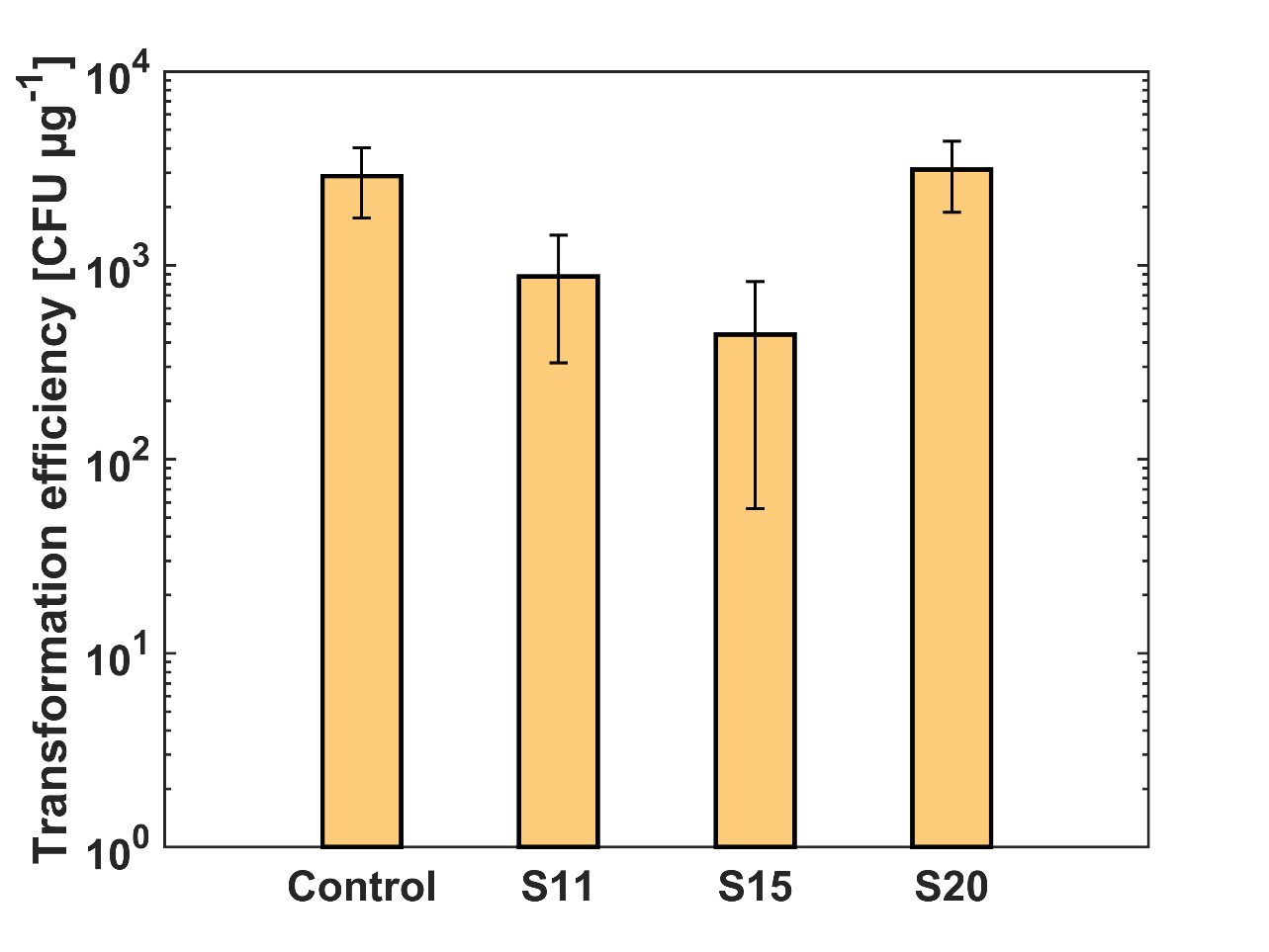


**Fig. S5** **Transformation efficiency of CRISPRi experiment.**

Transformation efficiency of shorter spacer plasmids (S11: 11-bp, S15: 15-bp, S20: 20-bp) targeting the pFAST gene integrated in the *ldh* locus. No significant difference in transformation efficiency with the control. Control: SPF-B017, empty vector. Data represent three biological replicates (average ± standard deviation).
